# Transcriptome Analysis Reveals Neuroprotective aspects of Human Reactive Astrocytes induced by Interleukin 1β

**DOI:** 10.1038/s41598-017-13174-w

**Published:** 2017-10-25

**Authors:** Daniel Boon Loong Teh, Ankshita Prasad, Wenxuan Jiang, Mohd. Zacky Ariffin, Sanjay Khanna, Abha Belorkar, Limsoon Wong, Xiaogang Liu, Angelo H. ALL

**Affiliations:** 1 Singapore Institute of Neurotechnology (SINAPSE), National University of Singapore, 28 Medical Drive, 5-COR, Singapore, 117456 Singapore; 2 Department of Biomedical Engineering, National University of Singapore, E4, 4 Engineering Drive 3, Singapore, 117583 Singapore; 3 Department of Orthopaedic Surgery, National University of Singapore, 1E Kent Ridge Road, Singapore, 119228 Singapore; 4 Department of Physiology, Yong Loo Lin School of Medicine, National University of Singapore, Singapore, Singapore; 5 Department of Computer Science, National University of Singapore, 13 Computing Drive, Singapore, 117417 Singapore; 6 Department of Chemistry, National University of Singapore, 3 Science Drive 3, Singapore, 117543 Singapore; 7 Department of Biomedical Engineering and Johns Hopkins School of Medicine, 701C Rutland Avenue 720, Baltimore, MD 21205 USA; 8 Department of Neurology, Johns Hopkins School of Medicine, 701C Rutland Avenue 720, Baltimore, MD 21205 USA

## Abstract

Reactive astrogliosis is a critical process in neuropathological conditions and neurotrauma. Although it has been suggested that it confers neuroprotective effects, the exact genomic mechanism has not been explored. The prevailing dogma of the role of astrogliosis in inhibition of axonal regeneration has been challenged by recent findings in rodent model’s spinal cord injury, demonstrating its neuroprotection and axonal regeneration properties. We examined whether their neuroprotective and axonal regeneration potentials can be identify in human spinal cord reactive astrocytes *in vitro*. Here, reactive astrogliosis was induced with IL1β. Within 24 hours of IL1β induction, astrocytes acquired reactive characteristics. Transcriptome analysis of over 40000 transcripts of genes and analysis with PFSnet subnetwork revealed upregulation of chemokines and axonal permissive factors including *FGF2, BDNF*, and *NGF*. In addition, most genes regulating axonal inhibitory molecules, including *ROBO1 and ROBO2* were downregulated. There was no increase in the gene expression of “Chondroitin Sulfate Proteoglycans” (*CSPG*s’) clusters. This suggests that reactive astrocytes may not be the main CSPG contributory factor in glial scar. PFSnet analysis also indicated an upregulation of “Axonal Guidance Signaling” pathway. Our result suggests that human spinal cord reactive astrocytes is potentially neuroprotective at an early onset of reactive astrogliosis.

## Introduction

Reactive astrogliosis is a common response of astrocytes to most Central Nervous System (CNS) injury^1,2^. The prevailing dogma of the involvement of reactive astrocytes in inhibition of axonal regeneration^3^, has been challenged by recent findings of Anderson *et al*. work, which demonstrated the critical role of reactive astrocytes in aiding axonal regeneration in rodents^4^. While some of the previous reports have identified reactive astrocytes as neuroprotective agents^1,5,6^, their direct neuroprotective effects were not well-documented in the human CNS astrocytes. As astrocytes’ functions differ among regions in the CNS^7^, we investigated the presence of axonal regenerating and neuroprotective properties of reactive astrocytes^4^ in human spinal cord derived astrocytes. Elucidating these properties at an early onset of reactive astrogliosis, will enable physicians and scientists to design novel therapeutic strategies exploiting the potential of reactive astrocyte mediated endogenous recovery.

It has been reported that among inflammatory mediators such as IL-1, TNF, IFN, and TGF, which are known to be active post-CNS injury, cytokine interleukin-1β (IL1β) is specifically critical for induction of reactive-astrocyte phenotype^8^. IL1β is a prominent inflammatory cytokine and is an early regulator of astrogliosis^9^. IL1β mRNA is highly up-regulated and persists 24+ hours after the onset of Spinal Cord Injury (SCI) at and around the epicenter of injury. This both initiates and modulates the inflammatory responses, leading to reactive astrogliosis^10^. Although bacterial endotoxin; lipopolysaccharides (LPS) could also be used for inducing reactive astrogliosis^11^, human astrocytes are unresponsive to LPS stimulation but are highly sensitive to IL1β^12^. In addition, it has also been reported that clusters of genes expression in reactive-astrocytes were highly dependent on various modalities of injury-induction^13,14^.

In our study, we used IL1β to induce reactivity in ***human spinal cord astrocytes*** *, in vitro* within 24 hours. Reactive astrogliosis is known to be invoked at the early phases of injury, with astrocytes acquiring a hypertrophic morphology and increased GFAP expression^15–17^ (Supp. Figure 1). IL1β treated human spinal cord astrocytes were found to undergo similar morphological transformations within 24 hours of exposure. Using transcriptome analysis, we have identified that *IL6, CXCL5* and *C15ORF48* (also known as *NMES1* gene) are the most up-regulated genes in human spinal cord reactive astrocytes. Whole genome transcriptome analysis shows changes in genes expression levels of 25 axonal growth permissive and 13 axonal growth inhibitory molecules. Particularly, the axonal growth promotion and neurotrophic factor genes like *BDNF* and *NGF* were upregulated. On the other hand, we detected no upregulation of *CSPGs* clusters of genes, which suggests that reactive astrocytes may not be the major contributors of CSPGs at the early onset (24 hours) of glial scarring. “Axonal Guidance Signaling” and “ECM-Receptor Interaction” pathways in reactive astrocytes, were differentially upregulates as compared to nascent astrocytes determined by PFSnet subnetwork analysis of differentially expressed genes (DEGs)^18^. Collectively, IL1β induced human spinal cord reactive astrocytes may exert various endogenous neuroprotective effects as demonstrated by the upregulation of critical axonal growth genes and downregulation of axonal inhibitory genes.

## Results

### Characterization of human spinal cord reactive astrocytes

**Figure 1:**
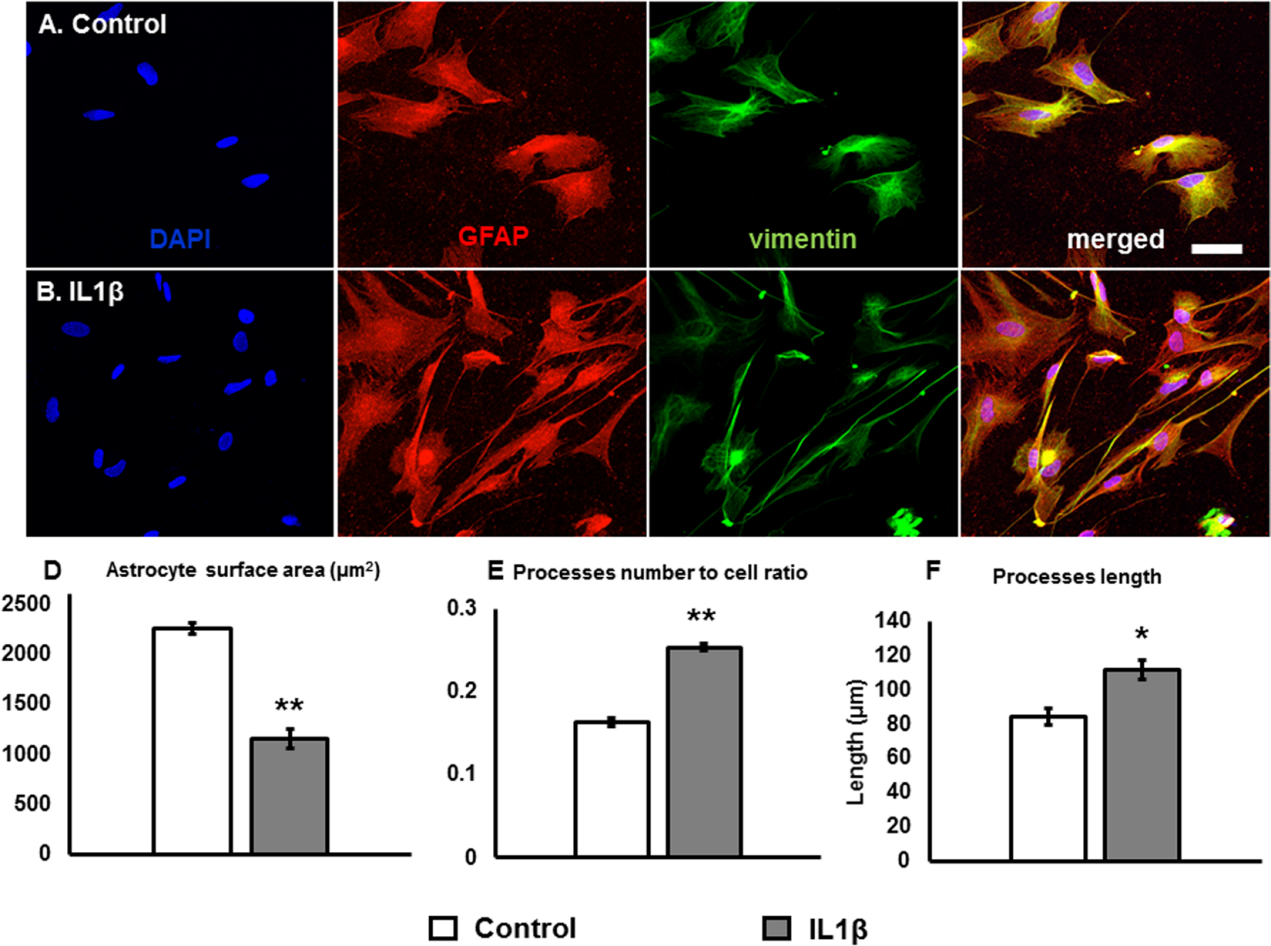
Human spinal cord astrocytes. (**A**) Control astrocytes stained with GFAP (red) and vimentin (green). **(B)** IL1β treated human spinal cord reactive astrocytes acquired more extensive processes. **(D)** The surface area was significantly reduced (*p* = *0.00863*) in reactive astrocytes as compared to control (1200 astrocytes counted for control and treated groups each). **(E)** The number of processes to cell ratio was significantly increased in reactive astrocytes (*p* = *9.09* × *10* ^*−6*^, 500 cells were counted each in control and treated groups) after induced by IL1β. **(F)** The processes length was significantly increased in reactive astrocyte as compared to control (*p* = *0.009*, 400 processes were counted for experiment and control each). Scale bar 50 μm.

We tested the homogeneity of the nascent human spinal cord astrocytes by staining with astrocyte markers: Glial Fibrillary Protein (GFAP) and vimentin **(**Fig. 1A)^19–22^. Prior to IL1β exposure, the astrocytes were 72 ± 2% positive for GFAP (4075 total cells counted in control group), while all the cells were vimentin^+^. 24 hours after exposure to 100 ng/ml of IL1β^23,24^; the astrocytes acquired bipolar shape and a shrunken morphology with extensive elongated processes **(**Fig. 1B). The average surface area of reactive astrocytes was reduced from 2262.6 ± 91 µm^2^ in control, to 1159.2 ± 52 µm^2^ in IL1β treated astrocytes **(**Fig. 1D). This change in the surface area was due to the fact that astrocytes acquired a more polarized morphology with extensive processes from the cell bodies. As reported in Fig. 1E, the number of processes to cell ratio for reactive astrocytes (0.25 ± 0) was increased in comparison to control group (0.16 ± 0). Although, a small fraction of control astrocytes displayed extensive processes, their lengths (84.6 ± 5 µm; *p* = *0.009*) were shorter as compared to IL1β treated astrocytes (111.9 ± 5 µm, 400 total processes counted for treated and experiment each) **(**Fig. 1F **)**.

### Genome wide analysis of human spinal cord reactive astrocytes

**Figure 2:**
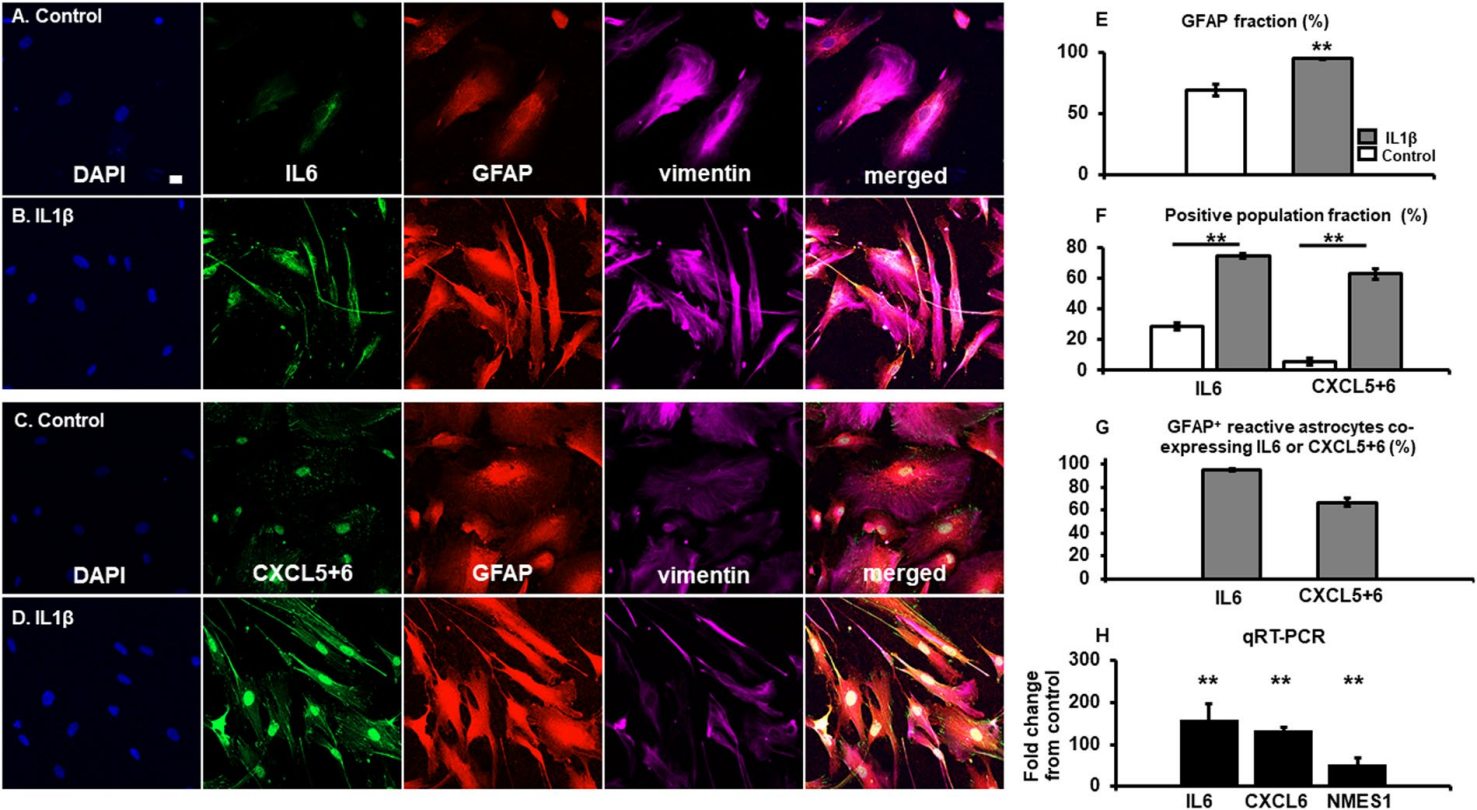
Reactive astrocytes. (**A**) Control astrocytes stained with IL6 (green), GFAP (red), vimentin (magenta), and DAPI (blue). **(B)** IL1β reactive astrocytes stained with IL6, GFAP, vimentin and DAPI. **(C)** Control astrocytes evaluated for CXCL5 + 6 immunocytochemistry, as compared to reactive astrocyte **(D)**. IL1β reactive astrocytes immunocytochemistry staining for CXCL5 + 6 (green). **(E)** The fraction of GFAP^+^ astrocytes population was significantly increased as compared to control. (*p* = *0.00152*, 2558 total cells counted in reactive astrocytes and 1962 total cells counted in control) **(F)** The overall population fraction positive for either IL6 (*p* = *3.4* × *10* ^*−6*^, 1299 cells counted in reactive astrocytes, and 965 cells counted in control) or CXCL5 + 6 (*p* = *0.00571*, 1059 cells counted in reactive astrocytes, 1297 cells counted in control) were significantly increased in reactive astrocytes as compared to control. **(G)** Within the GFAP^+^ cells, 94.9 ± 1% were also co-labelled with IL6, while 66.7 ± 4% were co-labelled with CXCL5 + 6 immunocytochemistry. **(H)** As a confirmation, a qRT-PCR was carried out for IL6, CXCL6 and NMES1, of which all were upregulated in comparison to control. IL6 (*p* = 7.01 × 10^−7^), CXCL6 (*p* = 2.34 × 10^−6^) and NMES1 (*p* = 6.4 × 10^−7^). The fold changes were respectively (IL6) 159.8 ± 37, (CXCL6) 53.0 ± 9 and (NMES1)133.1 ± 15 folds from control. Scale bar 20 μm. (n = 4 independent experiments).

To investigate reactive astrocytes mediated-endogenous neuroprotection and axonal regeneration potentials, genome wide analysis of reactive astrocytes was carried out. Supp. Figure 2 shows the scatter plot of differential expression genes between reactive astrocytes and nascent astrocytes. Table 1 shows non-discriminatively the 20 most upregulated genes while, Table 2 shows the top 20 genes downregulated 24 hours after inducing reactive-astrogliosis. Most prominently, *IL6* (223 folds)*, CXCL5* (205 folds), and *C15orf48* (also known as *NMES1*) (108 folds) were upregulated. *EPHA7* was the most downregulated gene, followed by *CEMIP* and *MTUS1* by −20, −18 and −18 folds, respectively. The complete list of changes in genes expression is provided in Supp. File 1. To elucidate whether GFAP^+^ or GFAP^-^ astrocytes were the main contributory factor in these transcriptome changes, the fraction of GFAP^+^ cells were first determined in control and in reactive astrocytes. The percentage of GFAP^+^ cells in reactive astrocytes was increased from 69.0 ± 5% in control to 94.6 ± 0% (*p* = *0.00152*) **(**Fig. 2A–D,E). The fraction of IL6^+^ population in reactive astrocytes were also increased from 28.6 ± 2% to 74.4 ± 1% **(**Fig. 2A,B,F **)** (*p* = *3.4* × *10* ^*−6*^). Likewise, CXCL5 + 6^+^ population fraction increased by more than 11 folds in reactive astrocytes from control **(**Fig. 2C,D,F) (*p* = *0.00571*). To determine the distribution of these IL6^+^ and CXCL5+ 6^+^ cells among GFAP^+/−^ reactive astrocytes, the fraction of IL6^+^/GFAP^+^ and CXCL5+ 6^+^/GFAP^+^ reactive astrocytes were tabulated. 94.9 ± 1% GFAP^+^ reactive astrocytes were also positive with IL6^+^, while 66.7 ± 4% of GFAP^+^ reactive astrocytes were also stained with CXCL5+ 6 **(**Fig. 2G). As a large percentage of GFAP^+^ astrocytes are double positive for IL6 or CXCL5+ 6 markers, the transcriptome changes can be considered to be a derivation from the GFAP^+^ reactive astrocytes. Additionally, qRT-PCR was carried out to confirm the increase in IL6 (*p* = 7.01 × 10^−7^), CXCL6 (*p* = 2.34 × 10^−6^) and NMES1 (*p* = 6.4 × 10^−7^). The fold changes were respectively (IL6) 159.8 ± 37, (CXCL6) 53.0 ± 9 and (NMES1)133.1 ± 15 folds from control **(**Fig. 2H).

**Table 1:** The 20 most upregulated genes in human spinal cord reactive astrocytes.

No.	Gene symbol	Fold induction	Description
1	*IL6*	223.01	interleukin 6
2	*CXCL5*	205.07	chemokine (C-X-C motif) ligand 5 (215101_s_at transcript id)
3	*CXCL5*	131.80	chemokine (C-X-C motif) ligand 5 (214974_x_at transcript id id)
4	*C15orf48*	108.20	chromosome 15 open reading frame 48 also known as *NMES1*
5	*CXCL2*	98.56	chemokine (C-X-C motif) ligand 2
6	*CXCL3*	67.81	chemokine (C-X-C motif) ligand 3
7	*CCL20*	65.40	chemokine (C-C motif) ligand 20
8	*CXCL8*	53.35	chemokine (C-X-C motif) ligand 8
9	*CXCL6*	49.07	chemokine (C-X-C motif) ligand 6
10	*CXCL1*	37.61	chemokine (C-X-C motif) ligand 1 (melanoma growth stimulating activity, alpha)
11	*CXCL5*	34.83	chemokine (C-X-C motif) ligand 5
12	*TNFAIP6*	19.95	tumor necrosis factor, alpha-induced protein 6
13	*IL1β*	18.38	interleukin 1 beta
14	*CSF2*	17.60	colony stimulating factor 2 (granulocyte-macrophage)
15	*MMP3*	17.51	matrix metallopeptidase 3
16	*BCL2A1*	17.06	BCL2-related protein A1
17	*CXCL8*	14.16	chemokine (C-X-C motif) ligand 8
18	*CSF3*	13.27	colony stimulating factor 3
19	*LOC285628*	13.20	MIR146A host gene
20	*AREG*	12.79	amphiregulin

**Table 2:** The 20 most downregulated genes in human spinal cord reactive astrocytes.

No.	Gene symbol	Fold induction	Description
1	*EPHA7*	−20.38	EPH receptor A7
2	*CEMIP*	−18.22	cell migration inducing protein, hyaluronan binding
3	*MTUS1*	−17.57	microtubule associated tumor suppressor 1
4	*EPHA7*	−17.05	EPH receptor A7
5	*COL14A1*	−14.93	collagen, type XIV, alpha 1
6	*MTUS1*	−14.67	microtubule associated tumor suppressor 1
7	*ACKR3*	−13.86	atypical chemokine receptor 3
8	*WNT2B*	−13.34	wingless-type MMTV integration site family, member 2B
9	*PI15*	−12.28	peptidase inhibitor 15
10	*SLC2A12*	−11.74	solute carrier family 2 (facilitated glucose transporter), member 12
11	*CXCL12*	−11.48	chemokine (C-X-C motif) ligand 12
12	*HTR2B*	−11.25	5-hydroxytryptamine (serotonin) receptor 2B, G protein-coupled
13	*TMEM178A*	−11.03	transmembrane protein 178 A
14	*COLEC12*	−10.56	collectin sub-family member 12
15	*RGCC*	−9.08	regulator of cell cycle
16	*SULF2*	−9.00	sulfatase 2
17	*SCD*	−8.90	stearoyl-CoA desaturase (delta-9-desaturase)
18	*LRRN1*	−8.88	leucine rich repeat neuronal 1
19	*SLC2A12*	−8.68	solute carrier family 2 (facilitated glucose transporter), member 12
20	*SESN3*	−8.63	sestrin 3

### Axonal guidance molecules and neurotrophic factors genes involved in neuroprotection and axonal regeneration

**Figure 3:**
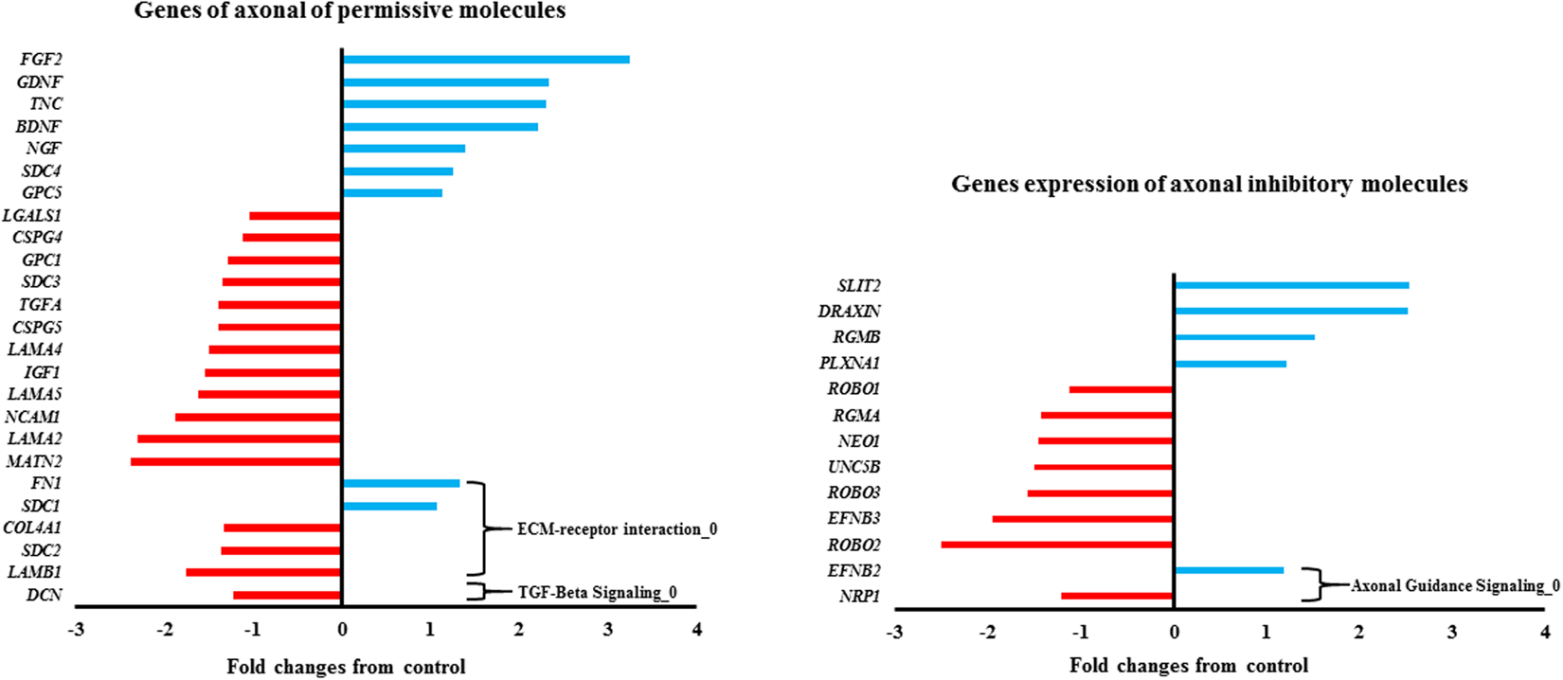
Gene fold expression of axonal permissive and inhibitory molecules in reactive astrocytes. 24 genes involved in axonal permissive molecules, were altered in reactive astrocytes from control. On the other hand, 8 out of 13 genes of axonal inhibitory molecules were downregulated. Differentially Expressed Genes that were listed in modulated biological pathways, were denoted with an arrow showing the biological subnetwork.

One of the aims of this study was to identify genes regulating axonal guidance molecules and neurotrophic factors expression that promote neuroprotection, neurogenesis and axonal regeneration in human spinal cord reactive astrocytes. Figure 3 shows the fold changes of these specific genes^4^. It is noteworthy that 9 out of 25 axonal growth permissive genes were upregulated in reactive astrocytes^25–41^. Of those, Fibroblast Growth Factor 2 (*FGF2)* is the most upregulated (3.24 folds), while matrilin2 (*MATN2)* (−2.37 folds) is the most downregulated axonal permissive genes. On the other hand, Slit Guidance Ligand (*SLIT2)* (2.54 folds) and Dorsal Inhibitory Axon Guidance Protein (*DRAXIN)* (2.52 folds) were the most upregulated genes involves in axonal growth inhibitory molecules. 8 out of 13 genes regulating axonal growth inhibitory molecules were down regulated, with Roundabout Guidance Receptor 2 (*ROBO2)* being the most downregulated genes in reactive astrocytes (−2.5 folds). Additionally, various matrix metallopeptidase and hyaluronan synthases were upregulated (Supp. File 1) as well. Interestingly, *CSPG*s clusters were unchanged in reactive astrocyte transcriptome as compared to control **(**Supp. File 1)^42–45^.

**Figure 4:**
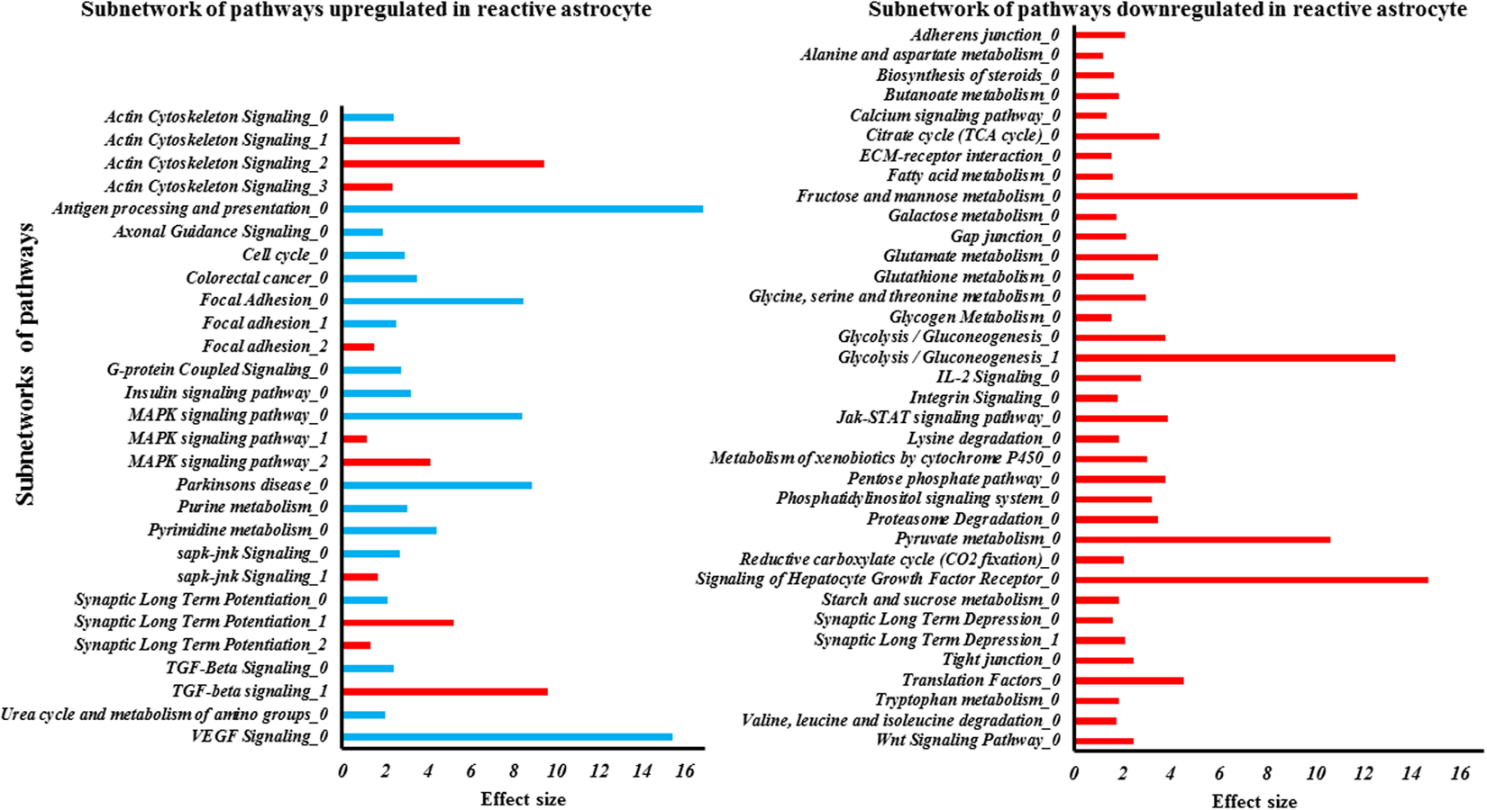
Subnetwork analysis in biological pathways in reactive astrocytes. Each major biological pathways are listed and the presence of different subnetwork pathways within a major biological pathway are defined by “0, 1, 2, 3, and 4”. Subnetworks are rank based on effect size. Blue graph indicates positively regulated, while red indicates negatively regulated.

Differential Expressed Genes (DEGs) were then analyzed with KEGG human pathways database to reveal affected biological pathways in human spinal cord reactive astrocytes. Using subnetwork analysis (PFSnet) within each pathway, it is revealed that reactive astrocytes differentially regulates subnetworks of various pathways, as compared to nascent astrocytes **(**Fig. 4 and Tables 3–4)^46^. Consistent with the observable changes in morphology of the *in vitro* reactive astrogliosis **(**Figs 1–2 **)**, PFSnet analysis revealed that actin cytoskeleton signaling pathway is one of the most altered pathway. As numerous pathways were differentially regulated in reactive astrocyte, our aim was to focus on main pathways that have critical role in axonal growth and development. We found that reactive astrocytes affect two subnetworks involved in axonal attraction and repulsion; *FYN* & *RASGAP* (Ras GTPase-activating protein 1) and *SFK* (*Src* family tyrosine kinase). In the *“Axonal Guidance Signaling”*, the axonal attraction and outgrowth seems to be regulated mainly by *FYN* and *RASGAP* **(**Supp. Figure 3). Another differential component of axonal attraction appeared to be directly regulated by *SFK*. In addition, a closely linked pathway to axonal development is the “ECM-receptor interaction” (Supp. Figure 4). Overall, the “ECM-Receptor Interaction” is downregulated in reactive astrocytes. Our result indicates that, fibronectin and laminin were downregulated, while collagen, Thrombospondin (THBS), and tenascin were upregulated.

**Table 3:** Lists of differentially expressed genes in each subnetwork of pathways.

Subnetworks in pathways	Effect size	Genes
Actin Cytoskeleton Signaling_0	2.44	*PDGFC, SH1, SGK1, GRB2, CDK2, MAP2K3, MAP2K, MAPK1, CDK4, CDK6, MAPK6, IRAK1, CSNK1A1*
Actin Cytoskeleton Signaling_1	5.49	*MYH*10*, PPP1CC, PPP1CA, MYH9, MYLK, MYL6, PPP1R12A, MYL6B*
Actin Cytoskeleton Signaling_2	9.44	*ARHGEF12, RAC1, NCKAP1, RHOA, IQGAP1, PIP4K2C, GNA13*
Actin Cytoskeleton Signaling_3	2.33	*RRAS, RRAS2, NRAS, PIK3R2, KRAS*
Antigen processing and presentation_0	16.87	*HLA-C, HLA-B, HLA-A, HLA-G, HLA-F, HLA-E*
Axonal Guidance Signaling_0	1.88	*EFNB2, ITGB1, PDGFC, CSNK1A1, SGK1, CDK4, SDC2, FYN, NRP1, MAP2K1, GRB2, VEGFA, PIK3R2, CDK6, MAPK6, MAPK1, RHOD, RASA1, SHC1*
Cell cycle_0	2.91	*PCNA, CCNB2, CCNB1, GADD45A, CDK4, CDK6, WEE1, YWHAG*
Colorectal cancer_0	3.47	*CCND1, MYC, MAPK1, JUN, MAP2K1*
Focal Adhesion_0	8.49	*ACTN1, ACTG1, RAC1, VCL, MYLK, ROCK1, MYL6, PPP1R12A, ACTB, RHOA, CDC42*
Focal adhesion_1	2.52	*PPP1CB, PPP1CC, PPP1CA, ROCK1, PPP1R12A, RHOA*
Focal adhesion_2	1.50	*ITGB1, ACTN1, PDGFC, ITGB5, SHC1, PRKCA, ITGB8, FYN, PDGFRA, ILK, MET, GRB2, VEGFA, PIK3R2, ACTG1, ACTB, PARVA, CAV2, VCL, CAV1*
G-protein Coupled Signaling_0	2.77	*PRKAR2A, SHC1, SGK1, ATF4, CREB3, CDK2, MAP2K3, MAP2K1, GRB2, CDK4, PRKAR1A, CDK6, MAPK6, MAPK1, IRAK1, CSNK1A1*
Insulin signaling pathway_0	3.21	*PPP1CB, CALM1, CALM2, PPP1CC, PPP1CA*
MAPK signaling pathway_0	8.42	*HSPA5, HSPA8, HSPA1A, MAPK1, MAPK6, CRK, MYC, DUSP1*
MAPK signaling pathway_1	1.14	*PRKCA, RRAS2, NRAS, RRAS, RASA1, KRAS*
MAPK signaling pathway_2	4.12	*MKNK2, MAP2K1, MAPK1, ATF4, MYC, DUSP1*
Parkinson’s disease_0	8.85	*SLC25A6, CYCS, SLC25A5, VDAC3, VDAC2, VDAC1, PPID*
Purine metabolism_0	3.07	*RRM2, RRM1, ADSL, HPRT1, NT5E, IMPDH2, POLR2H, PAICS, ENTPD4, POLR2E, POLR2G, GUK1, POLR2B, POLR2L, ADK, POLR2K, POLR2J, ATIC, NME1, NME2, AK2, POLE3, CANT1, DGUOK*
Pyrimidine metabolism_0	4.44	*RRM2, RRM1, CMPK1, DCTD, NT5E, DTYMK, ENTPD4, POLR2E, POLR2G, POLR2B, POLR2L, DUT, POLR2H, POLR2K, POLR2J, NME1, NME2, AK3, TYMS, ITPA, POLE3, CANT1*
sapk-jnk Signaling_0	2.68	*CSNK1A1, SGK1, MAPK6, CDK2, MAP2K3, MAP2K1, GRB2, CDK4, CDK6, CRK, MAPK1, IRAK1, SHC1*
sapk-jnk Signaling_1	1.66	*RRAS, KRAS, RRAS2, NRAS, RAC1*
Synaptic Long Term Potentiation_0	2.13	*CREB3, RAP1B, RAP1A, PRKAR2A, MAP2K1, MAPK1, PRKAR1A, ATF4*
Synaptic Long Term Potentiation_1	5.24	*RAP1B, RAP1A, PRKAR2A, MAP2K1, MAPK1, PRKAR1A, ATF4*
Synaptic Long Term Potentiation_2	1.35	*CALM2, CALM1, PRKCA, RRAS2, NRAS, RRAS, KRAS*
TGF-Beta Signaling_0	2.44	*CSNK1A1, SGK1, GRB2, MAP2K1, MAP2K3, CDK2, MAPK1, CDK4, CDK6, MAPK6, IRAK1*
TGF-beta signaling pathway_1	9.59	*DCN, TGFBR2, TGFB2, LTBP1, PPP2CA, PPP2CB, THBS1, RHOA*
Urea cycle and metabolism of amino groups_0	2.01	*SAT1, AMD1, SMS, ODC1*,
VEGF Signaling_0	15.45	*ACTA2, ACTG1, ACTG2, VCL, PRKAR2A, PRKAR1A, ACTB*

**Table 4:** Lists of genes for pathways downregulated in reactive astrocytes.

Subnetworks in pathways	Effect size	Genes
Adherens junction_0	2.12	*ACTN1, TJP1, ACP1, ACTB, RAC1, IQGAP1, VCL, FYN, CSNK2B, CTNNB1, MET, CDC42, PTPRF, RHOA, PTPRM, CTNNA1, CSNK2A1, ACTG1*
Alanine and aspartate metabolism_0	1.20	*DARS, GOT2, ASS1, ASNS, NARS*
Biosynthesis of steroids_0	1.66	*FDPS, IDI1, LSS, CYP51 A1, FDFT1, SQLE*
Butanoate metabolism_0	1.87	*ECHS1, ACAT2, HADHA, HSD17B4, ACAT1*
Calcium signaling pathway_0	1.34	*CAMK2D, PPP3CB, CALM2, MYLK, CALM1*
Calcium Signaling_0	4.89	*RAP2B, CALM2, CALM1, RAP1B, RAP1A, PRKAR2A, MAPK1, PRKAR1A, ATF4*
Citrate cycle (TCA cycle)_0	3.55	*IDH3B, IDH3G, DLD, IDH2, IDH1, ACO1, SUCLG1, MDH2, MDH1, DLST, CS, ACLY, PDHB, SDHA, PDHA1, SDHD, FH, SDHC*
ECM-receptor interaction_0	1.57	*LAMC1, COL11A1, FN1, CD44, SDC2, SDC4, THBS1, COL5A2, COL6A1, COL5A1, COL1A2, COL4A2, COL4A1, COL1A1, LAMB1, COL3A1, COL6A3, TNC, CD47*
Fatty acid metabolism_0	1.59	*ACADVL, ECHS1, HADHB, ACSL3, HADHA, ACAT1, ACAT2, HSD17B4*
Fructose and mannose metabolism_0	11.75	*PHPT1, HK1, TPI1, PFKP, PFKM, ALDOA*
Galactose metabolism_0	1.76	*AKR1B1, HK1, PGM1, UGP2, GLB1*
Gap junction_0	2.14	*GJA1, TJP1, TUBA1C, TUBA1B, TUBA1A, TUBB, TUBB3, TUBB6, MAP2K1, MAPK1, PRKCA, TUBB2A, TUBB2B*
Glutamate metabolism_0	3.50	*GLS, GLUD1, GLUL, GOT2, EPRS, QARS*
Glutathione metabolism_0	2.48	*GSTO1, GSTA4, GSTM3, TXNDC12, GPX1, MGST3, GSTP1, MGST1, GPX4*
Glycine, serine and threonine metabolism_0	3.00	*PSPH, SHMT2, PSAT1, PHGDH, SARS, GARS*
Glycogen Metabolism_0	1.53	*CALM2, CALM1, PPP2CA, PPP2CB, PGM1, PYGB*
Glycolysis / Gluconeogenesis_0	3.78	*PGM1, PFKM, GPI, HK1, GAPDH, PFKP, ENO1, TPI1, PGAM1, ALDOA, PGK1*
Glycolysis / Gluconeogenesis_1	13.34	*PDHB, PDHA1, LDHA, LDHB, DLD*
IL-2 Signaling_0	2.75	*PTPN11, GRB2, PIK3R2, SHC1, JAK1*
Integrin Signaling_0	1.82	*ACTA2, ACTG1, ACTG2, RHOQ, RND3, RHOC, RHOA, ACTB, RHOD*
Jak-STAT signaling pathway_0	3.88	*PTPN11, IL6ST, GRB2, PIK3R2, IFNGR2, JAK1, OSMR*
Lysine degradation_0	1.87	*ACAT1, ACAT2, HADHA, HSD17B4, ECHS1*
Metabolism of xenobiotics by cytochrome P450_0	3.02	*GSTO1, GSTA4, GSTM3, MGST3, GSTP1, MGST1*
Pentose phosphate pathway_0	3.78	*GPI, TALDO1, PGM1, PFKP, TKT, ALDOA, PFKM*
Phosphatidylinositol signaling system_0	3.24	*PTEN, CDIPT, PIK3R2, PIP4K2C, SYNJ2*
Proteasome Degradation_0	3.50	*PSME2, PSME1, PSMB7, PSMB6, PSMB5, PSMB4, PSMB3, PSMB2, PSMB1*
Pyruvate metabolism_0	10.63	*PDHA1, DLD, ME1, MDH2, MDH1, PDHB, LDHA, LDHB*
Reductive carboxylate cycle (CO2 fixation)_0	2.07	*IDH2, IDH1, FH, MDH2, MDH1, ACO1, ACLY*
Signaling of Hepatocyte Growth Factor Receptor_0	14.67	*MAP2K1, MAPK1, JUN, RAP1B, RAP1A*
Starch and sucrose metabolism_0	1.88	*GPI, UXS1, UGDH, HK1, PGM1, PYGB, GBE1, UGP2*
Synaptic Long Term Depression_0	1.62	*PPP1R7, PPP1R3C, PPP1CC, PPP1CA, PRKCA, PPP2CB, PPP2CA, MAP2K1, MAPK1, PPP1R12A, PPP1R14B*
Synaptic Long Term Depression_1	2.10	*YWHAZ, GNAS, PRDX6, GNA13, GRN, GNAI3*
Tight junction_0	2.48	*MYH*10*, TJP1, ACTG1, PRKCA, MYH9, CTNNA1, CTNNB1, CTTN, CLDN11, ACTB, RHOA*
Translation Factors_0	4.53	*EIF1, EIF1AX, EIF5B, EIF4H, EIF4B, EIF4A1, EIF4A2*
Tryptophan metabolism_0	1.87	*ACAT1, ACAT2, HADHA, HSD17B4, ECHS1*
Valine, leucine and isoleucine degradation_0	1.73	*ALDH7A1, ECHS1, ACAT2, HADHA, HSD17B4, ACAT1, HADHB, ALDH9A1*
wnt Signaling_0	0.98	*TCF4, TCF3, GJA1, CCND1, CD44, MYC*

## Discussion

In this study, we report the genome wide transcriptome profile of IL1β induced human spinal cord reactive astrocytes. We have studied a comprehensive subnetwork analysis based transcriptome pattern, which reveals the genes and their associated molecular function along with the important signaling pathways that regulate reactive astrogliosis. This provides a molecular roadmap to facilitate intervention and to optimize the benefits of reactive astrogliosis by either augmenting subnetworks of axonal attraction in the “Axonal Guidance Signaling” pathway or inhibiting signaling cascades in the “Axonal Repulsion” subnetwork.

*CXCL5* and *NMES1* are among the most upregulated genes by 205 and 108 folds respectively. The association of *NMES1* with human spinal cord reactive astrocytes is not well-known currently. We speculate that a very likely role of *C15orf48* or commonly known as *NMES1* may be in the regulation of the cell-cycle process, as its downregulation has been associated with potential tumorigenesis. Similarly, rat model of reactive-astrocyte using lipopolysaccharide (LPS, chemical injury) and middle carotid arterial occlusion (MCAO, ischemic injury) to induce injury did not identify *CXCL5* expression in their clusters of differentially expressed genes. Hence, we deduce that reactive astrocytes are a heterogeneous cell population whose molecular identity is based on the type of injury and origin of host cells^13,14,47–49^.

The changes in the “Actin Cytoskeleton Signaling” pathway can be attributed to the change of the cytoskeleton into the more diffuse and ring-structure actin filaments^50^, leading to morphology changes in reactive astrocytes^14^. The reorganization of the actin cytoskeleton is affected by inflammatory cytokines and is closely related to the “Focal Adhesion” and “ECM Receptor Interaction” pathways. A component of MAPK signaling pathway, ERK phosphorylation has been shown to be increased after SCI^51^. However, our PFSnet subnetwork analysis suggest that for the “MAPK signaling pathway”, the “*JNK* and p38 MAPK” sub-networks is activated in reactive astrocytes, while the classical “MAPK kinase sub-pathway” is down regulated. This selective subnetwork activation has not been clearly defined previously in reactive astrogliosis. Although the TGF-β signaling pathway is known to be involved in astrocyte reactivity, its exact signaling cascade has not been well-established in reactive astrocytes. Our findings indicate that one subnetwork of the pathway, involving *ERK* is upregulated while the other subnetwork involving the *RhoA* and *PP2A* is down regulated. Subnetwork analysis offers the advantage of differential view within a biological pathway, rather than revealing only an overall general alteration of biological pathway.

One intriguing observation was an increase in neurotrophic factors and “Axonal Guidance Signaling” pathway, particularly brain derived neurotrophic factor *(BDNF*) in human spinal cord reactive astrocytes. The other neurotrophic factor that was upregulated was nerve growth factor (*NGF*). These two factors are critical in development, survival and regeneration of axons. Our findings thus suggest that early onset of reactive astrocytes could potentially promote neuronal and axonal development. Reactive astrocytes in the glial scar thus have the potential to secrete multiple axon-growth-permissive molecules, thus improving the microenvironment at and around the epicenter of injury. This will facilitate neuronal repair and regeneration. Recent findings also suggest that BDNF regulates the development of oligodendrocyte precursor cells^4,52–56^. Our results suggest that the primary axonal attraction aspect of reactive astrocytes, could be attributed to *RasGAP* and *FYN* over expression. Additionally, we also report the upregulated of “VEGF signaling” pathway, which has neurotrophic and neuroprotective effects on neuronal cells^57^.

We also identified that there was an absence of up regulation of CSPGs genes. It has been known that CSPGs are major axonal growth inhibitor in glial scar. Our results present the possibility that reactive astrocytes may not be the main contributor of CPSGs at the epicenter and around the injury. Furthermore, we also reported the upregulation of hyaluronan synthases, which are enzymes for producing hyaluronan. The overexpression of hyaluronan is known to improve SCI recovery by reducing the lesion, and pro-inflammatory cytokines suggesting another neuroprotective properties of reactive astrocytes.

Modification of extracellular matrix is an important element in formation of glial scar and modulation of axonal growth, regeneration and neuronal development post-CNS injury. *MMP3, MMP1*, and *MMP12* belonging to the cluster of matrix proteins were found to be upregulated in reactive astrocytes. These genes have been associated with recruitment and migration of nascent astrocytes to the site of reactive astrocyte. Furthermore, *MMP3* and *MMP12* overexpression has been associated with remyelination. However, an increase in expression of matrix metalloproteases is known to enhance brain blood barrier (BBB) permeability and immune cells infiltration, resulting in inflammation post-CNS injury^58^. Our results indicate the upregulation of collagen and tenascin as well as downregulation of laminin and fibronectin in the ECM-receptor interaction pathways. In particular, collagen is critical in the early phase of tissue repair^59^ and the role of tenascin, in promoting neural outgrowth is controversial. Laminin and fibronectin are well-known for promoting neurite outgrowth. Collectively, we interpret that the ECM changes in reactive astrocytes may potentially induce both neuro-permissive and inhibitory effects to modulate axonal regeneration and growth^59–66^.

In our report, we presented a fetal human spinal cord reactive astrogliosis induced by IL1β. Although the genomic profiles of fetal astrocytes and adult astrocytes are comparable, notable differences in expression has been identified from previous literature^67^. This include higher expression of pro-inflammatory miRNAs expression in adult astrocytes as compared to fetal^68^, while lower expression of fetal germinal matrix miRNAs in adult astrocytes as compared to fetal^67^. The higher expression of matrix associated miRNA could reflect the migrating status of the developing astrocytes in fetal CNS. Furthermore, young astrocytes also expressed genes involved in “neuronal differentiation”^69^. As our study is based on reactive astrogliosis in fetal astrocytes, it remains to be explored if neuroprotective and axonal regeneration potentials can replicated in adult reactive astrocytes. Also, our analysis indicates that both neuroprotective and inhibitory genes are activated in reactive astrocytes post 24 hours of IL1β exposure, suggesting that a balance of these gene expressions may regulate the functionality of reactive astrocytes.

## Materials and Methods

### Cell Culture

Human astrocytes derived from the spinal cord (19 weeks old fetus) was purchased from ScienCell Research Laboratories (Cat. No: 1820)^3^. The cells were cultured in Astrocytes Basal Medium (ScienCell^TM^) supplemented with 2% FBS (ScienCell^TM^), 1% Astrocyte growth supplement (ScienCell^TM^) and 1% of penicillin-streptomycin (ScienCell^TM^). The human astrocytes cells were plated on Matrigel (Corning) coated polystyrene 35mm plates with cell density of 1 × 10^5^ cells. 24 hours after plating, cells were washed with PBS (HyClone™). Fresh medium containing IL1β (Invitrogen^TM^) at 100 ng/ml concentration was added and incubated for 24 hours in 5% CO2 at 37 °C.

### Immunohistochemistry

(i) *In vitro:* Human spinal cord astrocytes were grown on four wells matrigel coated dishes (Ibidi Inc.) at density of 2 × 10^4^. They were fixed with 4% paraformaldehyde (PFA) in PBS for 10 minutes at 4 °C. The fixative solution was removed and cells were washed three times with PBS for 5 min each at 4 °C. Cells were permeabilized and blocked with blocking buffer for 30 minutes at 4 °C. Primary antibodies (1:200; GFAP) [Millipore], (1:50; IL-6) [sc-1265 Santa Cruz], (1:50; Cxcl5 + 6) [ab198505, Abcam] and (1:250;Vimentin) [RD system] was diluted in blocking buffer and incubated overnight at 4 °C. Samples were then washed three times with washing buffer for 10 minutes each at 4 °C. Secondary antibodies were added (Alexa 488, Alexa 564 and Streptavidin 643 conjugated antibodies, 1:200) in blocking buffer and cells were incubated at room for 4 hours. Cells were then washed three times with washing buffer for 5 minutes each at room temperature. Nuclei were stained with DAPI for 10 min at room temperature. DAPI solution was aspirated and washed once washing buffer for 5 minutes at room temperature was performed. Mounting medium DAKO was added and the samples were incubated at 4 °C overnight.

### RNA isolation and microarray

Cells were collected after exposure to IL1β for 24 hours, Accutase (StemPro®) was added to detach the cells. RNA was extracted using RNeasy Mini Kit (Qiagen, CA, USA) per manufacturer specifications.

### Microarray

*(i)* All RNA samples were quality checked prior to microarray analysis in accordance to Affymetrix recommended protocols. Affymetrix 3′ IVT PLUS reagent kit was used. All RNA samples were assessed with spectrophotometric measurements (BioSPEC-Mini, Shimadzu) and quality RNA Integrity Number (RIN) with Agilent Bioanalyzer, *(ii)* for target preparation, 100 ng of total RNA was reverse transcribed to generate cDNA, and later used as template to generate biotin-labeled amplified RNA (aRNA). aRNA was then fragmented and hybridized to Affymetrix Human U133 Plus 2.0 Arrays, for 16 hours (45 °C; 60 rpm rotation). Affymetrix Human U133 Plus 2.0 Arrays (HG-U133 Plus 2.0) comprised of 1,300,000 unique oligonucleotides, encompassing 47, 000 transcripts and variants, representing approximately 39, 000 of well characterized human genes, providing a complete coverage to the human genes. All samples were processed with similar reagent kit, were washed, stained and scanned with Affymetrix 300 7 G scanner. Scanned images were assessed for hybridization efficiency. *(iii)* Prior to microarray analysis, quality control checks were also carried out on the array. The 3′/5′ ratio of housekeeping genes, presences of spike controls, background value, raw Q noise, scaling factor, and percent of genes presence were evaluated. Signal intensity ratio of 3′/5′ probe sets is important in establishing good cDNA synthesis, integrity of starting RNA and hybridization properties. In order to obtain reliable and accurate data comparison, the background intensities were measured and were made sure to be consistent and closed to each other. Additionally, the raw Q noise was also measured by performing pixel to pixel variations in background intensities. This is to make sure that the variations in the digitized signal observed by the scanners as it samples the probe array’s surface is consistent and close to each other. The measure of brightness of the array which may vary between arrays to array was also normalized to a standard level. This normalization will be important in comparing array to array data. To assess the hybridization, washing and staining steps quality, bacteria spike controls were performed. These are probe sets hybridized by pre-labeled bacterial spike controls (BioC, BioC, BioD, and Cre in staggered concentration). Finally, Poly A control was also carried out to identify problems with target preparation. These are a set of poly-adenylated RNA spikes of Lys, Phe, Thr, and Dap, prepared in staggered concentration. These are prepared together with the samples throughout cDNA synthesis onwards.

All RNA samples have RIN 10 and good quality absorbance (OD) ratio for 260/280 m, (>1.8) and 260/230 (>2.0) **(**Supp. Table 1, Supp Fig. 5), suggesting high purity of RNA, with minimal degradation. Furthermore, the 3′/5′ ratio of housekeeping gene GAPDH, approximated 1 onwards **(**Supp. Table 2 **)**. The array was also quality checked with consistent background intensity ranging from 30.781324 to 40.71225 **(**Supp. Table 2 **)**. The normalized scaling factors was done to standardize the measure of brightness of the array, and the result of the array falls within the acceptable range of below 3-fold **(**Supp. Table 2 **)**. In the bacteria spike controls, all samples also showed correct signal intensities of the control including BioB, suggesting good hybridization **(**Supp. Fig. 6). To rule out any problems with the target preparation, PolyA controls were also carried out, and all samples have shown consistent and staggered concentration of the poly A controls **(**Supp. Fig. 6).

### Analysis of array intensities

For the changes in differential genes expression level, Affymetrix Transcriptome Console was used to analyze the level of expression for each individual gene. One-way between subject ANOVA, was used to assess the statistical significance (*p* < *0.05*).

### Quantitative Reverse-Transcriptase Polymerase Chain Reaction

qRT-PCR was performed on ViiA™ 7 Real-Time PCR System (Applied Biosystems™) using SYBR Green PCR Master Mix reagent (Applied Biosystems ™). Specific PCR products for *IL-6* (Fwd-ATAGCCCAGAGCATCCCTCC, Rev-GGGTCAGGGGTGGTTATTGC), GFAP (Fwd-CAGATTCGAGGGGGCAAAAGC, Rev-AGGCTCACTCCCTGTCAAGC), and *Cxcl 6* (Fwd-TGCGTTGCACTTGTTTACGC, Rev-CTTCCCGTTCTTCAGGGAGG) *NMES1* (Fwd-GCCCACCAGGCGATCAATAC, Rev-ACACAGCGAAAGATGAGGCT) were detected with the fluorescent double-stranded DNA binding dye, SYBR Green. qRT-PCR amplification was performed in triplicates for each sample and the results were replicated in four independent experiments. Gel electrophoresis and melting curve analyses were performed to validate PCR product sizes. The expression level of each gene was normalized against β-actin using the comparative CT method^70^.

### Subnetwork analysis

Candidate subnetworks were generated by inducing connected components on known biological pathways with highly expressed genes in each phenotype. Two scores are computed for each subnetwork; these scores denote the level of expression of the subnetwork in majority of the samples in each phenotype. Finally, the difference of the two scores is tested for statistical significance. The theoretical *t*-distribution is used as the null distribution for estimating the statistical significance of subnetworks scored in PFSNet. An additional criteria set was that any subnetwork tested for statistical significance, has to be highly expressed in all the samples in the corresponding group (control/IL1β). Changed in subnetworks were analyzed with pathway information from the PathwayAPI database^34^ which contains the aggregation of human pathways from KEGG^71^ and Ingenuity. Expression data is preprocessed using the espresso function in R affy package^336^. Pathways maps were generated based on KEGG pathways database tool^3^. Each subnetwork was ranked by their effect size. Effect size is a quantitative measure of difference between two groups. In our study, the effect size for each subnetwork was computed as the standardized mean of paired difference “(mean of paired differences)/(standard deviation of paired differences)” between PFSNet scores, corresponding to the control and reactive astrocyte groups^72,73^. As the sample size is small, multiple-testing correction was not performed since the *p*-value of the same gene fluctuates in a small range, with a large portion in the insignificant part even though the gene is differentially expressed by construction. Multiple-testing correction approaches would simply shift the null-hypothesis rejection threshold left-wards and thus would be insensitive against the wide fluctuation range in the *p*-value of this gene. We have also discuss similar approach previously^62,63^.

### Imaging

The samples were imaged using ZEISS LSM 810 confocal microscopy instrument. Image J (Fiji) was used for image analysis and quantification. Tile scan and stitching were performed with ZEN blue software to image the entire spinal cord slice. Quantification for spinal cord GFAP intensity was done over 5 random Region of Interest (ROIs), covering the grey and white matter. The ROI is a 200 × 200 μm^2^ square visualized with (Plan-APOCHROMAT Zeiss NA = 0.45) 10X objective lens. 3 slices with 400 um between each of them were used for each rat. For *in vitro* cell length and processes quantification, 20X Plan-APOCHROMAT (NA = 0.8) objective lens was used. Cells that displayed processes were selected for quantification since not all the cells displayed a morphology that included processes. The main primary process of the cell was defined as any extension protruding from the cell body. The length of the primary process was defined as the distance from the center of the nuclei to the tip of the extended process. Only the main primary processes were used for quantification since very few cells displayed branching processes after exposure to IL-1B. For morphological quantification, the grayscale images were converted to binary type using ImageJ and their threshold was adjusted to clearly demarcate the cell boundary. The wand tool was used to outline the cellular perimeter in order to measure the cell surface area. Measuring was done using the line tool in Image J. Imaging settings were fixed for all groups within each experiments.

### Statistical analysis

All results were expressed as mean ± SEM, unless stated. Experiments were repeated independently for four times (n = 4) unless stated. Statistical significance was evaluated with unpaired student *t-*test for significance (*p < *0.05*; **p < *0.01*).

## Electronic supplementary material

Supplementary Data

Microarray array results
