# Supplementary material for: Transcriptome Analysis Reveals Neuroprotective aspects of Human Reactive Astrocytes induced by Interleukin 1β
Source: Sci Rep. 2017 Oct 25;7:13988. doi: 10.1038/s41598-017-13174-w (PMC5656635; doi:10.1038/s41598-017-13174-w)
Supplement: Supplementary file 1 — Supplementary Data [file 41598_2017_13174_MOESM1_ESM.doc]

**Supplementary Data: Transcriptome Analysis Reveals Neuroprotective aspects of Human Reactive Astrocytes induced by Interleukin 1β**

Daniel T. B. Loong^1^, Ankshita Prasad^2^, Jiang Wenxuan^3^, Mohd. Zacky Ariffin^4^, Sanjay Khanna^4^, Abha Belorkar^5^, Limsoon Wong^5^, Xiaogang Liu^6^†, Angelo H. ALL^1, 7, 8^*

**Affiliations:**

*^1^Singapore Institute of Neurotechnology (SINAPSE), National University of Singapore, 28 Medical Drive, 5-COR, 117456, Singapore. Email: lsidtbl@nus.edu.sg*

*^2^Department of Biomedical Engineering, National University of Singapore, E4, 4 Engineering Drive 3, 117583, Singapore. Email: ankshitaprasad@u.nus.edu*

*^3^Department of Orthopaedic Surgery, National University of Singapore, 1E Kent Ridge Road, 119228, Singapore. Tel: +65-6601 3198 Fax: +65-6873 3905*

***^4^****Department of Physiology, Yong Loo Lin School of Medicine, National University of Singapore. Emails:* [*phsmza@nus.edu.sg*](mailto:phsmza@nus.edu.sg)*, phsks@nus.edu.sg*

*^5^Department of Computer Science, National University of Singapore, 13 Computing Drive, Singapore 117417. Email: abhab@comp.nus.edu.sg, wongls@comp.nus.edu.sg.*

*^6^Department of Chemistry, National University of Singapore, 3 Science Drive 3, Singapore 117543, Singapore. Email: xiaogangliu@nus.edu.sg Tel:+65-6516 1352 Fax: +65-6779 1691*

*^7^Department of Biomedical Engineering and ^8^Department of Neurology, Johns Hopkins School of Medicine, 701C Rutland Avenue 720, Baltimore, MD 21205, USA. Tel: +410-502-5393*

† **Co-corresponding author**

* **Corresponding author email addresses:** [hmn@jhu.edu](mailto:hmn@jhu.edu), angelo.all@nuhs.edu.sg

**Supplementary Methods**

*In vivo:* All experimental procedures were performed in accordance and approved by the Institutional Animal Care and Use Committee (IACUC) of National University of Singapore. Six adult female Sprague-Dawley rats (200-220 grams) were used for two groups of control and injury. Rats had access to food and water *ad lib.*

*Laminectomy and Contusion Injury:* A 0.25ml mixed dose of ketamine and xylazine (75mg/kg; 10mg/kg body weight) were injected intraperitoneally to induce anesthesia for surgical procedures. To inflict SCI, laminectomy at T8 was performed to expose the dorsal surface of the spinal cord and T6 and T10 spinal processes were fixed to immobilize rats during the contusion. T8 was positioned 12.5mm under the rod of NYU-Impactor to induce moderate SCI. The velocity, impact time and trajectory of contusion were recorded to ensure consistency and reproducibility among all rats. The paravertebral muscle layers and skin were subsequently sutured. 24 hours after SCI, rats were administered an overdose of urethane (1.5g/kg i.p., Sigma) and transcardially perfused with ice cold 0.05M sodium nitrite (Sigma) followed by 4% paraformaldehyde (Sigma) in 0.1M phosphate buffer (Merck). The thoracic regions of spinal cords were extracted and fixed overnight at 4^o^C. A 3mm segment of the thoracic spinal cord containing the lesion site (T8 region) was isolated and sectioned with vibrotome (Leica VT1200S, Leica), as the length of an adult rat T8 spinal cord section is approximately 3mm. Consecutive 100µm thick sections were collected in 0.1M PBS. These slices were then used for immunohistochemistry.

*Immunohistochemistry:* Rat spinal cord slices (100µm) were fixed in PBS buffer containing 4% paraformaldehyde, for 30 minutes at 4ºC. Slices were then blocked in blocking buffer of PBS containing 5% normal donkey serum and 0.3% Triton X-100 overnight at 4ºC. Slices were then incubated with primary antibody of mouse anti-GFAP IgG (MAB360, Millipore) in blocking buffer, overnight at 4ºC. Slices were washed three times with washing buffer of PBS containing 0.1% Triton X-100 at 4ºC for 30 minutes each time. Donkey anti-mouse IgG conjugated to 546 was used to stain the primary antibody. Secondary antibody incubation was carried out at room temperature for 3 hours, while samples were shaking. Slices were washed three times of 30 minutes each, with washing buffer (PBS containing 0.1% Triton X-100) and stained with DAPI (1μg/ml). Slices were mounted on coverglass with DAKO mounting medium.


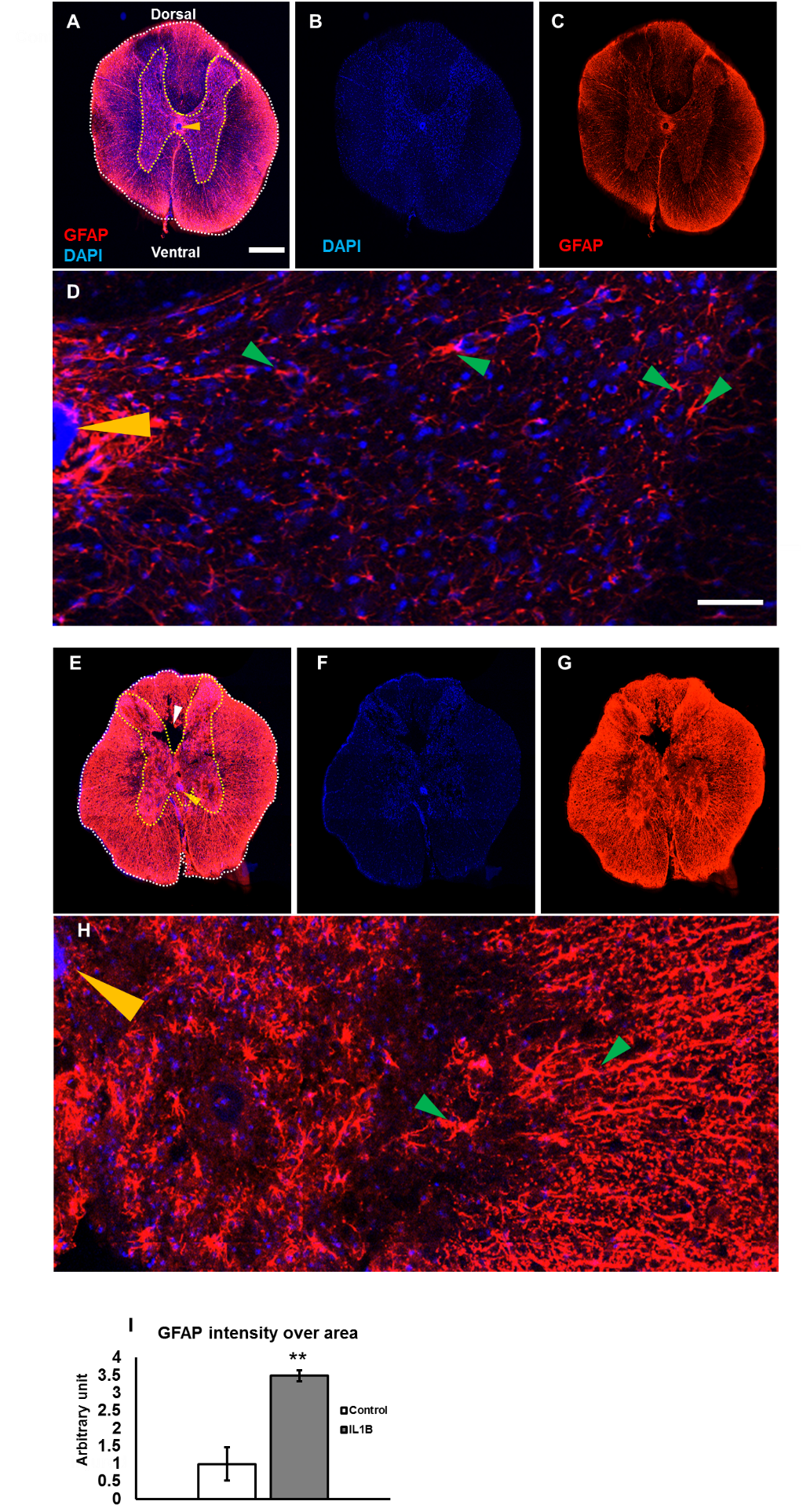
***Supplementary Figure 1* (A-C).** Spinal cord section from laminectomy only rats stained with GFAP (red) to mark astrocytes and DAPI (blue) for nucleus staining. **(A)** Yellow dotted lines show the border for the grey matter, while white dotted lines show the border for the entire spinal cord section. Orange arrows indicate the central canal. Scale bar = 500 μm **(D)** Magnified figure of **(A).** Astrocytes in non-injured rat spinal cord. The orange arrow shows the similar central canal in **(A)**, used as a reference point. Green arrow shows some of the GFAP^+^ astrocytes. Scale bar = 50 μm **(E-G)** 24 hours after a moderate contusion injury in the rat T8 spinal cord slice, GFAP intensity was significantly increased. **(E)** The white arrow shows the cavity formed due to the SCI. **(H)** Magnified figure of **(E).** Reactive astrocytes morphology were more extensive and hypertrophic as compared to control. Orange arrow indicates the central canal, similar to the location of **(E)**, and was used as a reference point. **(I)** The mean GFAP intensity in the histological examination. The mean GFAP intensity was 3.49±0.5 in contused spinal cord samples and 0.99±0.2 in control (laminectomy only slices) (***p=0.008;* N=3 rats/ group*)*. Images represent single optical section.

**
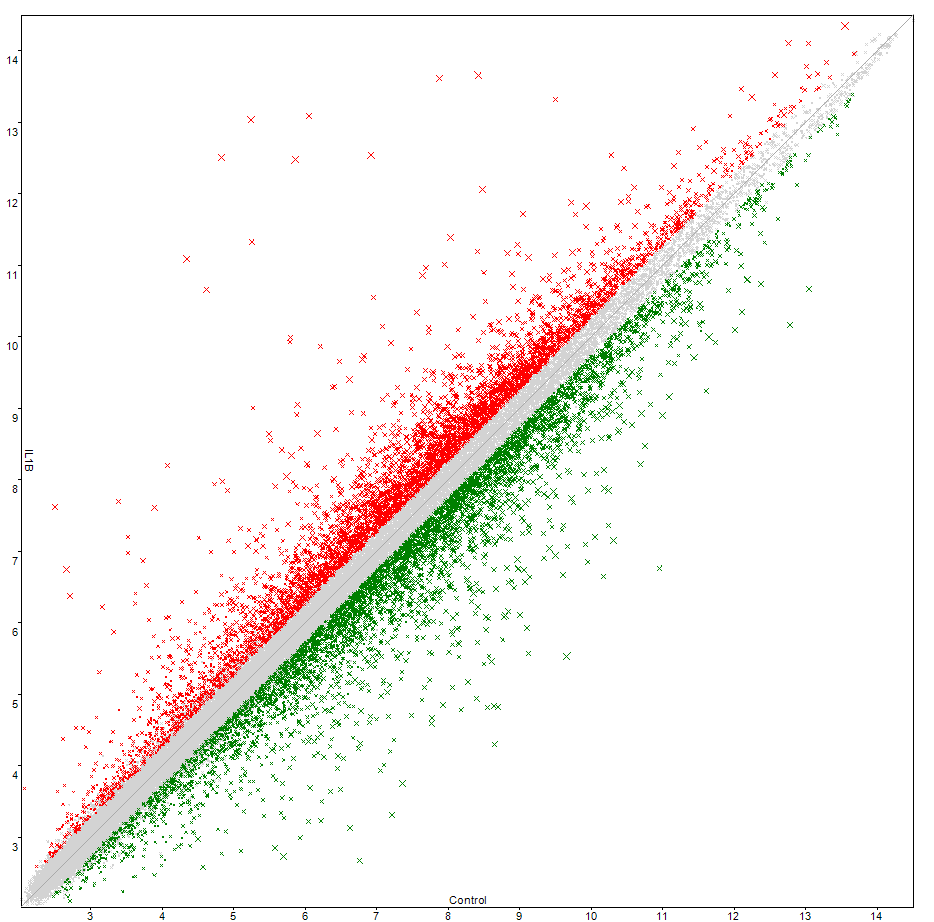
**

**Control**

**IL1β**

**Supplementary Figure 2.** Scatter plot of genes expression in human spinal cord reactive astrocytes and nascent astrocytes.

**Supplementary Figure 3. Axon guidance pathway**. Differential upregulation and down-regulation in human spinal cord reactive astrocyte, as compared to nascent astrocytes. Pathway was adapted from KEGG database^42^.


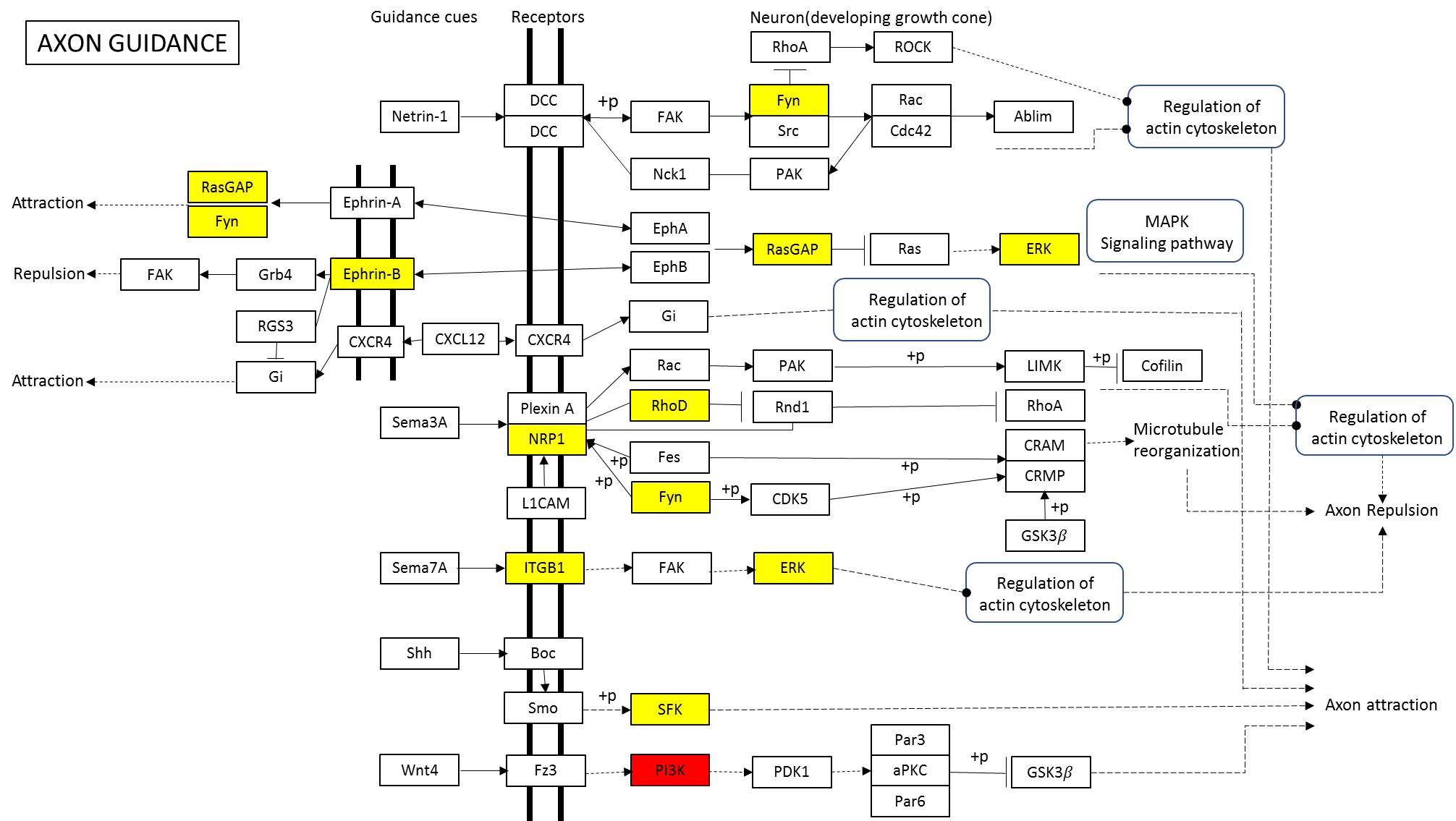

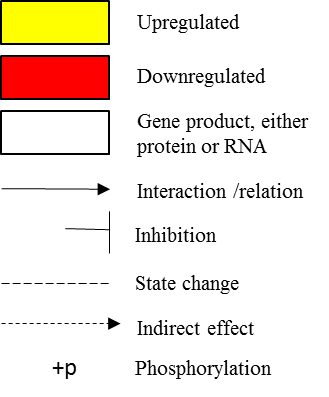


**Supplementary Figure 4. ECM-Receptor Interaction pathway**. Differential upregulation and downregulation in human spinal cord reactive astrocyte, as compared to nascent astrocytes. Pathway was adapted from KEGG database^42^.


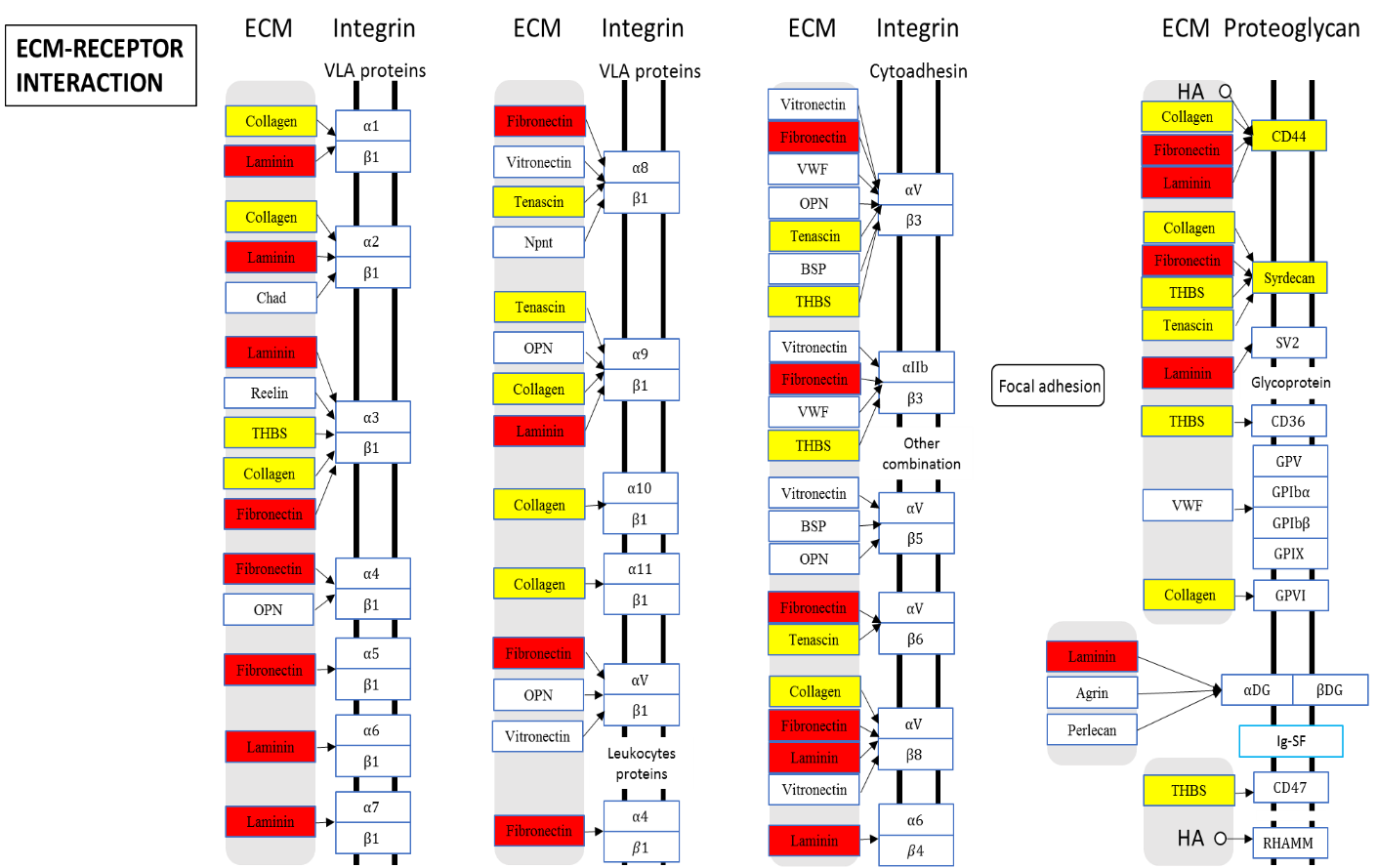

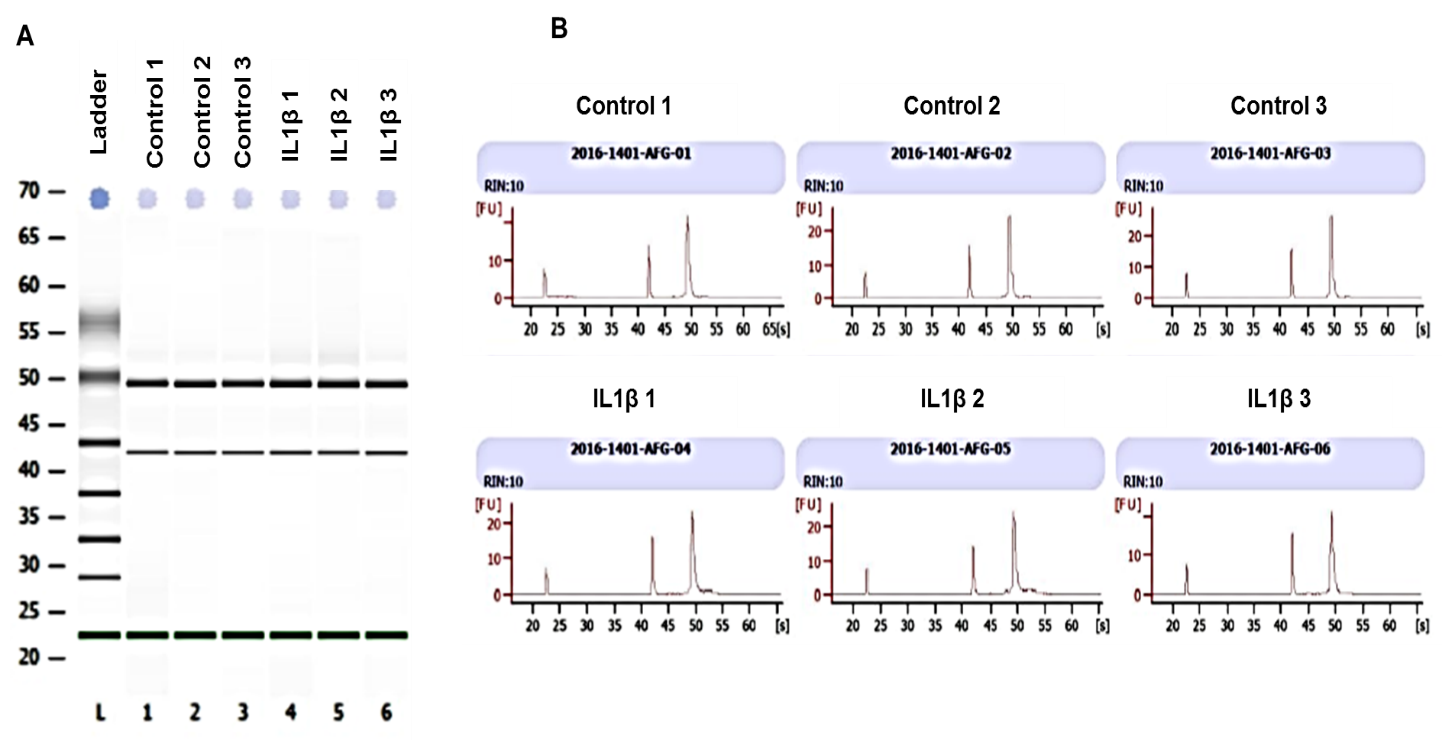


**Supplementary Figure 5. Quality control checks for RNA samples. (A)** Agilent Bioanalyzer image **(B)** Electrograms for all 6 samples. The result suggest no degradation of RNA samples.

**Supplementary Figure 6. Microarray quality control checks. (A)** **Average intensities of Poly A controls.** BioB, BioC, BioD, and Cre show increasing signal intensity including BioB, indicating good hybridization. **(B) Average intensities of Poly A controls.** Poly A controls are important to pinpoint problems with target preparations. All samples shown to have consistent staggering concentrations of the poly A controls (Lys<Thr<Phe<Dap). *Y*-axis represents arbitrary unit of intensity.


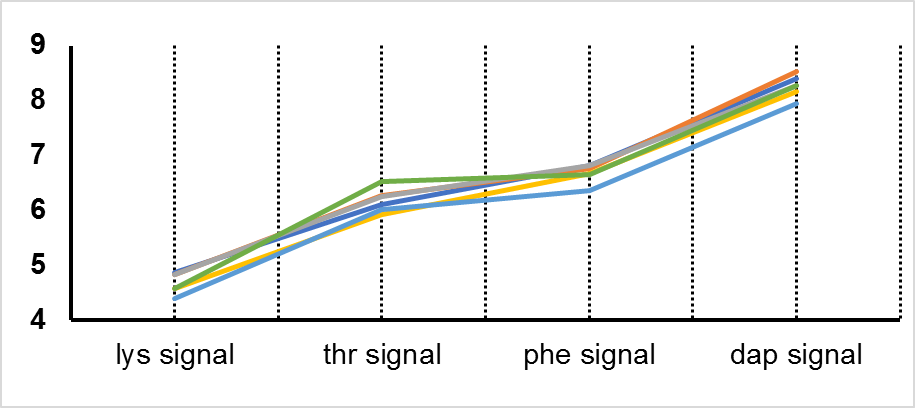

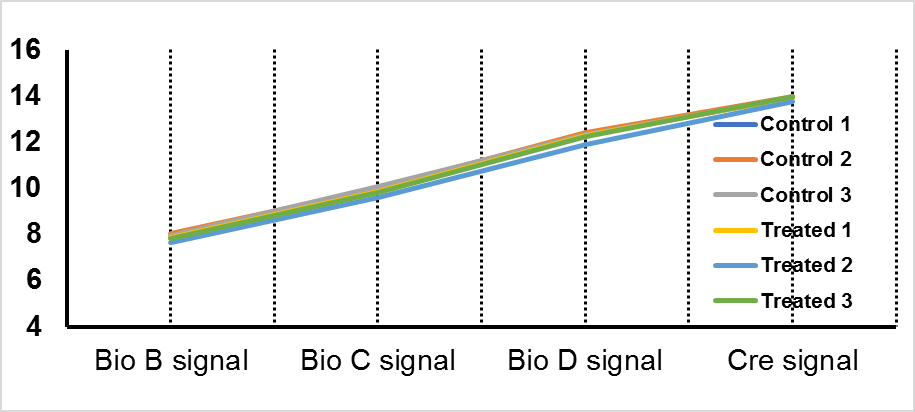


**Supplementary Table 1:** Spectrophotometer evaluation of RNA quality

| **Sample ID** | **OD 260nm** | **OD 280nm** | **OD 320nm** | **Ratio 260/280** | **Ratio 260/230** |
| --- | --- | --- | --- | --- | --- |
| **Control 1** | 0.6089 | 0.3126 | 0.0145 | 1.994 | 2.478 |
| **Control 2** | 0.5597 | 0.2925 | 0.0123 | 1.954 | 2.492 |
| **Control 3** | 0.5479 | 0.2854 | 0.0122 | 1.961 | 2.482 |
| **IL1β 1** | 0.7471 | 0.3845 | 0.0187 | 1.991 | 2.485 |
| **IL1β 2** | 0.8682 | 0.4445 | 0.0217 | 2.002 | 2.467 |
| **IL1β 3** | 0.7559 | 0.3875 | 0.0195 | 2.001 | 2.443 |

**Supplementary Table 2:** Array Quality Control Metrics

| **Sample ID** | **Scaling Factor** | **Raw Q** | **Background** | **%Percentage** | **3’/ 5’ GAPDH** |
| --- | --- | --- | --- | --- | --- |
| **Control 1** | 5.243395 | 1.048971 | 30.781324 | 43.518974 | 1.03 |
| **Control 2** | 4.556951 | 1.216705 | 33.819565 | 42.668495 | 0.993394 |
| **Control 3** | 5.155314 | 1.258249 | 33.397823 | 41.651577 | 1.034685 |
| **IL1β 1** | 4.087735 | 1.314023 | 38.752464 | 42.077732 | 0.997227 |
| **IL1β 2** | 3.807588 | 1.484244 | 40.71225 | 42.103336 | 0.97499 |
| **IL1β 3** | 3.928442 | 1.285192 | 38.018757 | 43.030636 | 1.021023 |
